# Mental healthcare services satisfaction and its associated factors among patients with mental disorders on follow-up in the University of Gondar Comprehensive Specialized Hospital, Northwest Ethiopia

**DOI:** 10.3389/fpsyt.2023.1081968

**Published:** 2023-05-30

**Authors:** Mamaru Melkam, Tilahun Kassew

**Affiliations:** Department of Psychiatry, College of Medicine and Health Science, University of Gondar, Gondar, Ethiopia

**Keywords:** mental healthcare services satisfaction, mental disorders, associated factors, satisfaction, psychiatry outpatients, Ethiopia

## Abstract

**Background:**

Patient mental healthcare services satisfaction is a crucial component in evaluating the effectiveness and efficiency of clinical service delivery. It can be explained as the client’s reaction to various aspects of the services they receive and their subjective assessment of the healthcare facilities and healthcare givers. Despite the importance of measuring mental healthcare services satisfaction, few studies have been conducted in Ethiopia. This study aimed to assess the prevalence of mental healthcare services satisfaction among patients with mental disorders who were on follow-up at the University of Gondar Specialized Hospital, Northwest Ethiopia.

**Method:**

An institution-based cross-sectional study was conducted from June 1, 2022 to July 21, 2022. All the study participants were interviewed on the follow-up visit consecutively. The Mental Healthcare Services Satisfaction Scale tool was used to measure patient satisfaction, and the Oslo-3 Social Support Scale and other questionnaires, such as environmental factors and clinical factors, were also screened. The data were checked for completeness, entered and coded using Epi-data version 4.6, and exported to Stata version 14 software for analysis. Bivariable logistic and multivariable regression analyses were employed to identify the factors significantly associated with satisfaction. Adjusted odds ratio (AOR) with a 95% confidence interval (CI) was used to report the result at a *p*-value of <0.05.

**Results:**

A total of 402 study participants were included in this study, with a response rate of 99.7%. The proportions of male and female participants who were satisfied with the mental healthcare services were 59.29 and 40.70%, respectively. The overall mental healthcare services satisfaction was 65.46% with a 95% CI of 59.90, 70.62. Not being admitted to psychiatry [AOR: 4.94; 95% CI (1.30, 8.76)], getting their drugs in the hospital [AOR: 1.34; 95% CI (3.58, 8.74)], and having strong social support [AOR: 6.40; 95% CI (2.64, 8.28)] were significantly associated with satisfaction.

**Conclusion:**

The prevalence of mental healthcare services satisfaction is very low; therefore, more is expected to be done to enhance the satisfaction of the patients who access these services via psychiatry clinics. Enhancing the social support of clients, making drugs available in the hospital, and improving the service received by the admitted client are necessary to increase the healthcare service satisfaction of clients on the whole. The services delivered in psychiatry units must be improved to achieve good patient satisfaction, which might be helpful for the improvement of the disorders.

## Introduction

Patient healthcare service satisfaction is one of the main areas identified for determining performance in the government’s National Service Framework for Mental Health (1). Mental health institutions have had to make sure that their patients are satisfied with the care they receive and how they are treated as clients since patients have taken on more of a consumer role in their healthcare (2). Despite initial concerns about the ability of people with mental illnesses to meaningfully express their inner thoughts and preferences, self-reported satisfaction ratings in individuals with mental disorders have repeatedly been demonstrated to be accurate and important assessments (3). People with mental illness are believed to experience lower service satisfaction than the population as a whole (4).

The degree of satisfaction experienced by patients while receiving mental healthcare services determines how satisfied they are with those services. According to the patient’s qualities, the quality of the care provided, or both, is expressed as the patient’s psychological response to the services they receive from the caregivers (3, 5). Patients who are satisfied with the outcomes of interventions are more likely to adhere to their treatment plans and make better use of available resources (6). Patients who use mental health services are more likely to participate in treatment and support their recovery when they are highly satisfied (6). However, it can be difficult to ensure that patients with different requirements are satisfied with their mental healthcare because in some cases, admission might be conducted involuntarily for their benefit.

In the past 20 years, there has been an increase in research on patient satisfaction with healthcare services since it is a reliable measure of the caliber of both individual healthcare and service delivery. There is a significant gap between the mental health service delivery and the patient’s satisfaction with mental health services (5, 7, 8). Different studies have reported that, globally, patients’ satisfaction with mental healthcare services ranges from 39.3 to 91.9% (9–12). Patients’ satisfaction differs across countries. For instance, the mental healthcare services satisfaction of patients with mental disorders was 90% in a study conducted in Ireland (12); a low satisfaction level (57%) was reported in the Indian study (10), and 66% of Nigerian patients with schizophrenia had good mental healthcare services satisfaction. Moreover, in Ethiopia, the reports showed that 44.5%–72% of patients with mental disorders had good mental healthcare services satisfaction levels (11, 13, 14), which shows that there is a discrepancy across different healthcare settings and at different periods.

Lower patient satisfaction affects clinical outcomes such as poor treatment adherence, which again leads to high treatment failures and relapse rates (8). The quality of life and self-management abilities in the treatment of a mental illness are impacted by low patient satisfaction (15). It also affects patients’ strong commitment to actively participate in their treatment plans and management of illness. This leads to an increase in the chances of re-occurrence of the symptoms (16). In addition, poor patient satisfaction relates to an increased risk of medical malpractice either from staff low competence or negligence that results in poor retention and adherence to their treatments (17). According to clinical diagnostic criteria, in this study, mental disorders, including psychotic anxiety, depression, and bipolar disorder, were incorporated into the mental healthcare services satisfaction.

Patient healthcare service satisfaction is associated with sociodemographic characteristics such as age, sex, living status, residence, and socioeconomic status (7, 11). Factors associated with poor patient satisfaction and mental healthcare services satisfaction include longer illness duration, having a comorbid health condition, schizophrenia diagnosis, early age of illness onset, relapse history, adherence to prescribed medication, and an increased number of prescribed medications (18–20). The quality of services from health institutions can affect the patient’s experience, for example, due to elements such as waiting time, the safety of the waiting area, busy personnel, cleanliness of the environment, physician’s competence, privacy, and confidentiality (21, 22). This centered on the idea of satisfaction coming from services given rather than individual characteristics. This concept includes both medical and non-medical aspects of health institution services. Having poor social support and the use of psychoactive substances also negatively affect patients’ mental healthcare services satisfaction levels (23, 24).

Patient satisfaction has been given significant weight in healthcare reforms that attempt to enhance the quality of health systems. In Ethiopia, few health institutions offer mental healthcare services by specialized professionals, which affects patients’ satisfaction with mental healthcare services. Patient satisfaction and related elements of mental health treatments were not studied previously at the University of Gondar Comprehensive Specialized Hospital in Northwest Ethiopia. Moreover, this study incorporated and examined the relevant clinical and psychosocial factors, which were not considered in the previous studies. Thus, this study aimed to determine the prevalence and associated factors of good satisfaction with mental healthcare services among patients with mental disorders attending psychiatric follow-ups at the University of Gondar Compressive Specialized Hospital, Northwest Ethiopia.

## Methods

An institution-based cross-sectional study was conducted on patients with mental disorders, attending psychiatric follow-ups at the University of Gondar Comprehensive Specialized Hospital, Northwest Ethiopia, from June 1, 2022 to July 21, 2022. The psychiatry unit is one of the departments that provide services in both outpatient and inpatient settings for patients with mental health disorders. On average, 820 patients have follow-up visits per month in the psychiatry outpatient clinic. Outpatients with mental disorders (psychotic, bipolar, and major depressive disorders and anxiety disorders) were diagnosed by the Diagnostic and Statistical Manual of Mental Disorders, Fifth Edition (DSM-5). The criteria were interviewed using a systematic random sampling technique. Patients aged 18 years and older who were on psychiatric follow-ups for at least 6 months during data collection were included. Patients with severe mental disorders (e.g., major depression and bipolar I disorder with psychotic features and schizophrenia) who were too ill to communicate were excluded.

### Sampling technique and sample size determination

Based on the single population proportion formula, the minimum sample size was calculated using a formula to estimate the proportion of patient mental healthcare services satisfaction by taking a proportion (*p* = 61.2%) from a previous study conducted at Dessie Specialized Hospital, east Ethiopia (9), standard normal distribution (Z = 1.96) with a confidence interval of 95% and *α* = 0.05, and margin of error (*d*) = 0.05.


$$\begin{array}{l} n=\frac{{\left(\frac{Z\alpha }{2}\right)}^{2}p\left(1-p\right)}{d^{2}}={\left(1.96\right)}^{2}\;\left(0.612\right)\;\left(0.388\right)/{\left(0.05\right)}^{2}\\ \;=364.8\sim 365 \end{array}$$

Using the single population proportion formula, the sample size is found to be 365.

After adding 10% for the likelihood of non-respondents, the final calculated sample size was 365 + 37 = **402**. We calculated the double proportion formula, but the sample size was lower than calculated by the single proportion formula; therefore, we took the large sample size.

#### Sampling procedure

Patients who have follow-up visits at data collection time were included in this study. The study participants, from whom the inclusion criteria are fulfilled, were interviewed according to the order of arrival. The respondent was interviewed in the waiting area before and after the main treatments at the outpatient department. The study respondents completed the informed consent before they were interviewed for the main questionnaire. The interview was held until the intended sample size was reached.

### Study variables

#### Dependent variable

✓ Patient satisfaction with mental healthcare services was poor/good.

#### Independent variable

✓ **Sociodemographic factors:** Age, sex, marital status, education status, occupation status, religion, residency, monthly income, and living circumstances.
✓ **Environmental-related factors:** Waiting time, distance from the hospital, availability of transport to the health institution, cost of transport, availability of drugs in the hospital, and cleanliness of the hospital.
✓ **Psychosocial and spiritual factors:** Social support, religious involvement, additional use of holy water, and additional use of cultural or traditional medicine.
✓ **Clinical factors:** Types of diagnosis, history of relapse, history of admission, duration of treatment, type of treatment, and the number of drugs taken.
✓ **Behavioral factors:** Current and past use of one of the following three substances: alcohol, khat, and cigarettes.

### Data collection procedures and tool

The questionnaire first was prepared in English, then translated into Amharic, and back-translated into English by language experts and mental health specialists. Training was given to all data collectors and supervisors on the data collection procedures for 2 days before the actual data collection. Closed supervision was carried out during the data collection time. The investigators checked the consistency of the filled questionnaire at the end of each day. The data were collected through face-to-face interviews using structured and standardized questionnaires. The questionnaire consisted of five parts.

The **first part** of the questionnaire was the sociodemographic characteristics of the study participants, which were developed from different literature.

The **second part** of the questionnaire was psychosocial and environmental factors of the study participants, which included the Oslo-3 Social Support Scale, a self-reported measure that consists of three items that ask about the number of close confidants, the sense of concern from other people, and the relationship with neighbors, with a focus on the accessibility of practical help. This tool is a three-item scale and has a range value of 3–14, further categorized as follows: “poor support,” 3–8; “moderate support,” 9–11; and “strong support,” 12–14. The Oslo-3 social support scale has been used in numerous studies, and these have confirmed the feasibility and predictive validity of the study mentioned in reference (25).

The **third part** of the questionnaire was mental healthcare satisfaction, which was measured using the Mental Healthcare Services Satisfaction Scale (MHSSS). Each statement on the scale includes four response options, as follows: strongly agree, agree, disagree, and strongly disagree. There is a total of 24 items on the scale that can be used to measure satisfaction with service. Each option has been assigned a number value, ranging from 1 for strongly disagree to 4 for strongly agree. The overall score is between 24 and 96, with a high value denoting high levels of satisfaction and a low value denoting low levels of satisfaction. According to the studies conducted in Ethiopia using this tool (MHSSS), the cutoff point is to conclude whether a patient’s satisfaction with mental healthcare services is good or poor. The mean score of the tool in this study was 64 (26).

The **fourth part** was the clinical factors of the study participants, including the current diagnosis, the total duration of illness, and others. It also includes the condition of the hospital waiting area and its distance from the home. The **fifth part** of the questionnaire was about the use of substances by the study participants, which includes alcohol, khat, and tobacco in this study.

### Operational definition

**Mental Healthcare Services Satisfaction:** This was screened using a Mental Healthcare Services Satisfaction Scale (MHSSS) measurement tool, measuring the service satisfaction of mental health patients. The score ranges from 24 to 96, and individuals with a total score lower than the mean score (64 for this study) had good patient satisfaction, and study participants who scored greater than or equal to 64 had poor satisfaction with the service (26).

**Social support:** This was measured using the Oslo-3 Social Support Scale. The sum score ranges from 3 to 14, with a high value representing strong levels and a low value representing poor levels of social support. In this document, social support was operationalized into three broad categories as follows: 3–8, poor social support; 9–11, moderate social support; and 12–14, strong social support.

**Current use of a substance**: This was operationalized as using at least one of a specific substance within the last 3 months (alcohol, khat, and tobacco).

### Data processing and analysis

The data were entered using Epi-Data version 3.1 and analyzed using Stata version 14 software. Descriptive statistics were presented using frequency and percentage to summarize the distribution of the data. The results were presented using tables. Binary logistic regression analysis was used to identify the factors associated with patient mental healthcare services satisfaction. Bivariable and multivariable logistic regression models were applied. Variables with a *p*-value of less than 0.2 at the bivariable analysis were entered into a multivariable logistic regression analysis (27). In the multivariable logistic regression analysis, an adjusted odds ratio (AOR) with a 95% confidence interval (CI) and a *p*-value of less than 0.05 has been used to determine statistically significant associated factors.

### Ethics consideration

Ethics approval was obtained from the University of Gondar institutional review board. A letter of permission was obtained from the University of Gondar College of Medicine and Health Science. Written consent from each study subject was asked for and secured after a detailed explanation of the main purpose of the study. The written informed consent for those not able to read and write was attained by a third person other than the data collector reading the statement and the participant providing their fingerprint. Confidentiality of the information forwarded by the subjects was assured by omitting the name of the study subject from the questionnaire, and a large effort was made to maintain the privacy of participants during the data collection time. The procedures we used in this research were comfortable for the patients. We also informed the patients in the consent document that they had the full right to participate, partially participate, or not to participate in the study, as well as to withdraw at any time.

## Result

### Sociodemographic characteristics of study participants

A total of 401 study participants were included in this study, with a response rate of 99.7%. Participants in this study had an average age of 41.08 years, with a standard deviation of ±12.9. Of the study respondents, 209 (52.1%) were male and 199 (49.6%) were married. More than half of the respondents (66.8%) were Orthodox believers, while 226 (56.4%) were from urban regions. Of the participants, 125 (31.1%) had a high school diploma, and 159 (39.5%) in the study’s sample lived with their spouses (Table 1).

**Table 1:** Sociodemographic characteristics of the study participants among psychiatry outpatients in the University of Gondar Compressive Specialized Hospital.

Variables	Category	Frequency	Percent
Sex	Male	209	52.1
	Female	192	47.9
Age	18–30	90	22.50
	31–40	119	29.75
	41–50	90	22.50
	>50	101	25.25
Marital status	Single	108	26.9
	Married	199	49.6
	Divorced	51	12.7
	Widowed/Er	20	5.0
	Separated	23	5.7
Monthly income in birr	<1,000	109	27.2
	1,000–5,000	189	47.1
	>5,000	103	25.6
Level of education	Unable to read and write	86	21.4
	Read and write	41	10.2
	1–8	64	16.0
	9–12	125	31.2
	Diploma and above	85	21.2
Religion	Orthodox	268	66.8
	Muslim	107	26.7
	Catholic	10	2.5
	Protestant	14	3.5
	Other*	2	0.5
Occupational status	Jobless	39	9.7
	Employee	103	25.7
	Housewife	42	10.5
	Self employed	90	22.4
	Farmer	73	18.2
	Merchant	33	8.2
	Other	21	5.2
Residence	Urban	226	56.4
	Rural	175	43.6
Living circumstance	Alone	58	14.5
	Parents	146	36.4
	Spouse	159	39.7
	Sibling	18	4.5
	Other**	20	5.0

Other* Pagan; Other** levied with friends and non-relatives.

### Clinical, psychosocial, and environmental factors

Of the respondents, 145 (36.7%) had received a diagnosis of schizophrenia, and 122 (40.5%) were solely taking antipsychotic medications. The majority of the participants (328, 81.8%) started to experience mental illness between the ages of 19 and 45 years. The average length of illness for 197 (47.9%) study participants was 1–3 years, and 169 (42.1%) had a history of recurrence. Of the participants, 202 (50.4%) and 292 (72.8%) had previously been hospitalized and had only recently been admitted. One-tenth (9.8%) of the respondents used psychoactive substances at one time (Table 2). Most of the study participants 285 (71.1%) practices religious prayer for their illness and only 49 (12.25%) used concurrent traditional medicine. Nearly half (46.6%) of the participants took holy water for their ailment at the same time, and more than half (54.9%) had moderate social support. Of the study’s participants, 346 (86.3%) experienced wait times of under 60 min, and 248 (61.8%) traveled less than 30 km at a cost of under 80 Ethiopian birrs (Table 3).

**Table 2:** Clinical and substance characteristics of study participants among psychiatry outpatients in the University of Gondar Specialized Hospital.

Current diagnosis	Schizophrenia	145	36.7
	MDD	109	27.6
	Bipolar disorder	71	18.0
	Anxiety	55	13.9
	Other	15	3.8
Number of medications	One drug only	296	73.8
	Two and more drugs	105	26.2
Currently on	Antipsychotics	122	40.5
	Antidepressants	113	37.5
	Mood stabilizer	22	7.3
	Anxiolytics	44	14.6
Age at onset of mental illness	Before 18	15	3.7
	19–45	328	81.8
	After 45	58	14.5
Total duration of illness	<1 year	57	14.2
	1–3 years	192	47.9
	4–6 years	48	12.0
	>7 years	104	25.9
History of relapse	Yes	169	42.1
	No	232	57.9
History of admission	Yes	202	50.4
	No	199	49.6
Availability of drugs in hospital	Yes	357	89.1
	No	44	10.9
Number of admissions	Once	292	72.8
	Twice	67	16.7
	Three and more	42	10.5
First contact with treatment	Religious	139	41.4
	Cultural	34	10.1
	Modern	163	48.5
Comorbid medical illness	Yes	92	23.0
	No	308	77.0
Current use of substance	Yes	39	9.8
	No	360	90.2
Ever use of substance	Yes	173	43.2
	No	227	56.8

**Table 3:** Psychosocial and environmental factors of the study participants among psychiatry outpatients in the University of Gondar Specialized Hospital.

Religious attendance prayer	Yes	285	71.1
	No	116	28.9
Concurrent use of holy water	Yes	187	46.6
	No	214	53.4
Concurrent use of traditional medicine	Yes	049	12.2
	No	351	87.8
Concurrent use of holy water	Yes	187	46.6
	No	214	53.4
Social support	Poor	118	29.4
	Moderate	220	54.9
	Strong	63	15.7
Waiting time in minutes	<60	346	86.3
	60–120	52	12.9
	>120	3	0.7
Easy to get transportation	Yes	385	96.0
	No	16	4.0
Distance	Under 30 km	248	61.8
	30–60 km	98	24.4
	Above 61 km	55	13.7
Cost transport	80 birr and below	207	60.2
	81–180	81	23.5
	Above 180 birrs	56	16.3

### The magnitude of patient mental healthcare services satisfaction

The percentage of patients who were satisfied with their mental healthcare services at the University of Gondar Specialized Hospital was 65.46%, with a 95% confidence interval (CI) of 59.90 and 70.62. The proportions of male and female participants who were satisfied with the mental healthcare services were 59.29% and 40.70%, respectively.

### Factors significantly associated with patient satisfaction

From bivariable logistic regressions, age, sex, level of education, current diagnosis, age of onset, total duration of illness, history of admission, cost of transport, strong social support, and availability of drugs at the public pharmacy were the variables associated with patient mental healthcare services satisfaction, with a *p*-value of less than 0.2. From multivariable logistic regressions, no history of admission, availability of drugs in the hospital, and having strong social support were the factors significantly associated with patient mental healthcare services satisfaction.

Patients who had no history of admission were five times more likely to have good mental healthcare services satisfaction compared to patients who had a history of admission [AOR = 4.94; 95% CI: 1.30–8.76]. The odds of mental healthcare services satisfaction were 1.34 times higher in patients who get their prescribed drugs in the hospital than those who bought their prescribed drugs from the private clinic [AOR = 1.34; 95% CI: 3.58–8.74]. Patients with mental disorders who had strong social support had seven times [AOR = 6.40; 95% CI: 2.64–8.28] higher odds of good mental healthcare services satisfaction compared with patients who had poor social support (Table 4).

**Table 4:** Bivariable and multivariable logistic regression tables for associated factors in the University of Gondar Specialized Hospital.

Variables	Category	Satisfactions		COR	AOR
		Yes	No		
Sex	Male	118	44	1	1
	Female	81	61	2.68 (1.89, 3.79)	0.56 (0.22, 1.40)
Age	18–30	48	16	2.80 (1.38, 5.66)	0.20 (0.25, 1.65)
	31–40	52	34	1.42 (0.78, 2.60)	0.43 (0.72, 2.57)
	41–50	54	13	3.87 (1.85, 8.10)	2.06 (0.35, 11.87)
	>50	45	42	1	1
Level of educations	Unable to read/write	33	35	1	1
	Able to read/write	17	10	1.80 (0.72, 4.49)	1.31 (0.15, 3.00)
	1–8	36	19	2.00 (0.96, 4.17)	1.31 (0.15, 3.00)
	9–12	71	16	4.70 (2.28, 9.68)	1.11 (0.18, 6.73)
	Diploma and above	42	25	1.78 (0.893.54)	2.30 (0.29, 7.81)
Current diagnosis	Schizophrenia	68	51	1	1
	MDD	59	25	1.71 (0.94, 3.10)	0.99 (0.012, 7.68)
	Bipolar disorder	34	17	1.24 (0.63, 2.44)	0.50 (0.05, 5.03)
	Anxiety	26	6	5.17 (1.7, 15.67)	0.92 (0.10, 8.21)
	Somatic symptoms	8	4	1.47 (0.42, 5.178)	0.28 (0.40, 2.29)
History of admission	Yes	75	80	1	1
	No	124	25	5.29 (3.10, 9.01)	4.94 (1.30, 8.75)*
Age of onset of illness	Before 18 years	7	6	3.97 (0.71, 5.15)	0.59 (0.142.39)
	19–45 years	160	81	3.55 (0.59, 4.35)	0.37 (0.93, 1.51)
	>45 years	32	18	1	1
Total	<1 year	35	6	0.47 (0.18, 1.21)	4.38 (0.44, 4.28)
Duration of illness	1–3 years	105	38	0.19 (0.06, 0.57)	4.71 (0.82, 2.68)
	4–6 years	18	16	0.15 (0.05, 0.409)	1.14 (0.22, 5.73)
	≥7 years	41	45	1	1
Availability of drug	Yes	8	30	0.07 (0.03, 0.19)	1.34 (3.58, 8.74)*
	No	191	75	1	1
Cost of transport	≤80 birr	138	36	0.20 (0.11, 0.37)	0.62 (0.12, 3.19)
	81–180 birr	30	38	0.24 (0.12, 0.46)	0.19 (0.25, 1.47)
	>180 birr	25	27	1	1
Social support	Poor	31	52	1	1
	Moderate	126	42	5.09 (2.85, 8.85)	3.14 (0.91, 3.16)
	Strong	42	11	6.40 (2.88, 4.02)	6.40 (2.64, 8.28)**

*p-value < 0.05; **p-value < 0.001.

## Discussion

This study aimed to assess the magnitude and factors associated with the mental healthcare services satisfaction of patients with mental disorders attending psychiatric follow-ups at a University of Gondar Compressive Specialized Hospital in Northwest Ethiopia. Patient satisfaction factors are used in mental health services because they have been linked to improving hospital administrative procedures and quality care. In the outpatient psychiatry clinic, the magnitude of mental healthcare satisfaction was 65.46%, with a 95% confidence interval (CI) of 59.90 and 70.62.

This result is consistent with that of earlier studies carried out in Dessie, Ethiopia (61.2%) (9), and Norway (28). However, this finding is lower than studies carried out in Pakistan (72%) (29), Sweden (77%), and Pakistan (90.7%) (29). The discrepancy may be caused by the use of different measurement tools that were locally developed, the sociodemographic characteristics of the study participants, and various methodological approaches that were used in other studies, as opposed to the interview techniques used in this study. In contrast, the result of this study was greater than those of studies carried out in Addis Ababa, Ethiopia 50.3% (30), and Qatar 55% (31). The discrepancy may be caused by the effect of varying severity in the patients’ mental disorders and the use of various measurement tools.

The majority of the variables linked to patients’ satisfaction with mental health services in this study were distinct from those in earlier research in Ethiopia. The previous research, for instance, did not evaluate and link the diagnosis of bipolar and psychotic illnesses, admission history, and the availability of prescribed medications in the community pharmacy. Having no prior history of hospitalization is statistically strongly correlated with satisfaction with mental health services. This result is consistent with studies carried out in Norway (28). The presence of involuntary admission to the hospital may be the cause of patients’ dissatisfaction with the mental healthcare they receive, which is a possible explanation for this link (28).

Another significant factor that is associated with patients’ satisfaction with mental healthcare services is the availability of drugs in the hospital. This finding is consistent with other studies conducted in Ethiopia and Morocco (30, 32). The possible reason for the association could be the lack of medications in hospitals, which may be the source of patients’ unhappiness since patients cannot afford or obtain the required prescriptions from a private pharmacy (30). The absence of medications at public health facilities, the high cost of psychotropic medications, and a lack of support from non-governmental organizations for long-term access to psychiatric medications throughout the spectrum of psychiatry service settings may further contribute to the association (33). Psychotropic medications are more expensive in private pharmacies than in public ones in Ethiopia, which discourages patients and leaves them dissatisfied with the level of service they receive.

Strong social support is another important factor that is significantly linked to satisfaction with mental healthcare services. This finding is in line with studies from Jimma, south Ethiopia (13), and Egypt (34). By accompanying patients to the hospital, purchasing necessary prescriptions, and reminding patients to take their prescribed medications as directed, strong social support may contribute to improved service. Offering emotional support at home is crucial, which will improve the patient’s functionality and treatment outcome as well as their perception of the quality of the care they receive (13). The impact of feeling helpful and receiving emotional support from family, friends, and other socially supportive others may also be considered as further supporting evidence.

This study’s main flaw may be social desirability bias, which occurs when people answer questions on patients’ satisfaction, substance usage, disease duration, and medication compliance in face-to-face interviews in a way that is more socially acceptable. The mental healthcare services satisfaction scale is a screening tool rather than a diagnostic one that can affect magnitude estimates. In addition, the research could not show the cause–effect relationships between factors and outcomes owing to its cross-sectional nature.

## Conclusion

In conclusion, the magnitude of the mental healthcare services satisfaction of patients with mental disorders in the University of Gondar Compressive Specialized Hospital in Northwest Ethiopia was low. The availability of the prescribed medication in the hospital, having no history of admission, and having strong social support were found to be the factors that enhance the patient’s mental healthcare services satisfaction. To increase the degree of satisfaction with healthcare services among patients with mental disorders, interventions targeted at identifying patients’ social support and making it easier for patients to access psychotropic medicines in the hospital are required. Follow-up studies are also recommended to assess the cause–effect relationships between patients’ levels of satisfaction, history of hospitalization, availability of medications in the hospital pharmacy, and social support with satisfaction in a local setting.

## Data availability statement

The original contributions presented in the study are included in the article/supplementary material, further inquiries can be directed to the corresponding author.

## Ethics statement

The studies involving human participants were reviewed and approved by University of Gondar Ethical approval commitee. The patients/participants provided their written informed consent to participate in this study. Written informed consent was obtained from the individual(s) for the publication of any potentially identifiable images or data included in this article.

## Author contributions

MM was involved in conceptualizing the study, study design, analysis, interpretation, report, and manuscript writing. TK made a significant contribution to the idea, study design, analysis and interpretation of the data, the writing of the text, and the revision of the manuscript for key intellectual elements. All authors contributed to the article and approved the submitted version.

## Conflict of interest

The authors declare that the research was conducted in the absence of any commercial or financial relationships that could be construed as a potential conflict of interest.

## Publisher’s note

All claims expressed in this article are solely those of the authors and do not necessarily represent those of their affiliated organizations, or those of the publisher, the editors and the reviewers. Any product that may be evaluated in this article, or claim that may be made by its manufacturer, is not guaranteed or endorsed by the publisher.

## Abbreviations

AOR: adjusted odds ratio
COR: crude odd ratio
CI: confidence interval
MHSSS: Mental Health Service Satisfaction Scale
OPD: Out-Patient Department
UGCSH: University of Gondar Comprehensive Specialized Hospital
